# Aggregating behaviour in invasive Caribbean lionfish is driven by habitat complexity

**DOI:** 10.1038/s41598-018-37459-w

**Published:** 2019-01-28

**Authors:** Christina L. Hunt, George R. Kelly, Hannah Windmill, Jocelyn Curtis-Quick, Helen Conlon, Max D. V. Bodmer, Alex D. Rogers, Dan A. Exton

**Affiliations:** 1 Department of Zoology, University of Oxford, John Krebs Field Station, Wytham, Oxford, OX2 8QJ UK; 2 Operation Wallacea, Wallace House, Old Bolingbroke, Spilsby, Lincolnshire PE23 4EX UK; 3 Department of Ocean and Earth Science, University of Southampton, National Oceanography Centre, Southampton, SO14 3ZH UK; 4 School of Earth and Ocean Sciences, University of Cardiff, Main Building, Park Place, Cardiff, CF10 3AT UK; 5 School of Environment, Earth and Ecosystem Sciences, The Open University, Walton Hall, Milton Keynes, MK7 6AA UK

## Abstract

Caribbean lionfish (*Pterois* spp.) are considered the most heavily impacting invasive marine vertebrate ever recorded. However, current management is largely inadequate, relying on opportunistic culling by recreational SCUBA divers. Culling efficiency could be greatly improved by exploiting natural aggregations, but to date this behaviour has only been recorded anecdotally, and the drivers are unknown. We found aggregations to be common *in situ*, but detected no conspecific attraction through visual or olfactory cues in laboratory experiments. Aggregating individuals were on average larger, but showed no further differences in morphology or life history. However, using visual assessments and 3D modelling we show lionfish prefer broad-scale, but avoid fine-scale, habitat complexity. We therefore suggest that lionfish aggregations are coincidental based on individuals’ mutual attraction to similar reef structure to maximise hunting efficiency. Using this knowledge, artificial aggregation devices might be developed to concentrate lionfish densities and thus improve culling efficiency.

## Introduction

Since their introduction in the early 1980s^1^, lionfish (*Pterois volitans* and *P. miles*) have spread throughout the western Atlantic with devastating impacts on the region’s coral reefs^2^. Successful eradications of marine invasive species are rare and generally involve species with a small invaded range^3,4^. Complete eradication of lionfish is considered unlikely^5^, as they are now well established with a large geographical distribution^6^ and have the potential for long distance dispersal^7^. Rather than eradication, management should therefore be focused on population reduction through culling^8^.

Lionfish culling is typically performed by recreational SCUBA divers and snorkelers using hand nets and pole spears^8^. This can be opportunistic or as part of organised events involving competing teams of divers and fishermen^9^. On shallow reefs, culling has been successful at reducing lionfish densities^8,10^ and allowing native species to recover^11^. However, whilst culling is largely restricted to depths <30 m^12^, lionfish are known to have invaded deeper mesophotic and sub-mesophotic habitats to at least 304 m^12,13^. Technical diving provides a partial solution, although cost, expertise and safety concerns will limit the extent of its use^14^, and lionfish traps to collect lionfish from deeper water are still being tested^15^.

Groups of lionfish have been observed throughout the invaded range^10,16,17^ and it has been noted that aggregating individuals often appear larger than solitary individuals^16,17^. By exploiting this natural behaviour and artificially stimulating aggregations, culling or trapping efficiency could be improved. Mass trapping of invasive and pest species has been successful in both aquatic and terrestrial ecosystems^18–20^ but requires knowledge of the species’ aggregation drivers to inform the design of effective lures to trap them. Although previous work has focused on many aspects of the lionfish invasion^21^, nothing is known about the drivers of lionfish aggregations, and quantitative data on their prevalence is lacking.

One possible driver of lionfish aggregations is social attraction, which may be for cooperative hunting (as observed in *P. miles* in its native range^22^), spawning^23^, minimising the chance of predation^24^ or pooling experiences to solve problems^25^. Social attraction in fish can be initiated by visual^26^, olfactory^27^, acoustic^26^ or a combination of cues^28^. Zebrafish will even aggregate with robotic fish that mimic their shape and movement^29^. Another possible aggregation driver is habitat preference. Fish may seek out specific habitat because it provides a source of prey, good hunting conditions or shelter from predators^30^. Marine habitat complexity can be measured via a range of metrics (e.g^31,32^) and is known to be a strong predictor of fish abundance^33,34^. Although lionfish have been anecdotally reported to be found in more complex habitat^10,16,17^, this has not been determined quantitatively.

Here, we provide the first quantitative assessment of lionfish aggregations and their drivers, specifically exploring: (i) morphology and life history traits of aggregating and solitary individuals via *in situ* population surveys and *ex situ* dissections, (ii) social attraction using laboratory binary choice tests focusing on visual and olfactory cues, and (iii) habitat preference and the role of habitat complexity using 3D modelling and visual scores.

## Results

### Aggregating lionfish are larger and heavier, but otherwise similar to solitary individuals

A total of 283 lionfish were caught and dissected for this study, comprising 178 solitary and 105 aggregating individuals. We recorded 33 aggregations of varying sizes from 2 to 7 individuals, with a mean (±1 SE) of 3 (±0.34) and a median of 2. Lionfish were collected from depths of 3.0–18.4 m, with similar mean depths (±1 SE) for solitary lionfish (10.7 ± 0.28 m) and aggregations (10.6 ± 0.52 m).

Lionfish found in aggregations were significantly larger than solitary individuals (*p* < 0.001) and were also heavier for their size (*p* < 0.001). Mean total lengths (±1 SE) were 22.2 cm (±0.55) for aggregating lionfish and 18.4 cm (±0.54) for solitary lionfish. However, there was no difference in sex ratio (*p* > 0.05), the gonadosomatic indices of males and females (*p* > 0.05 for both) or percentage body fat (*p* > 0.05) between solitary and aggregating individuals.

### Lionfish are not attracted to conspecific visual or olfactory cues

The proportion of trials where lionfish entered the stimulus zone first did not differ significantly from chance levels for any of the stimuli tested (*p* > 0.05 for all stimuli; Table 1). Lionfish in the visual, visual + olfactory and artificial model treatments showed no significant preference for either stimulus or control (*p* > 0.05 for each; Table 1). Lionfish in the olfactory treatment showed a significant preference for the control, or avoidance of the stimulus, with a mean (±1 SE) time difference of −229 (±62) seconds.

**Table 1:** Results of laboratory social attraction experiments.

Stimulus	Sample size	Initial preference		Overall preference	
		Proportion of trials where stimulus zone was entered first	*p*-value	Test statistic	*p*-value
Visual	15	0.6	0.6072	NA	0.6072
Olfactory	13	0.62	0.5811	−3.6764	0.00317*
Visual + olfactory	18	0.28	0.09625	−0.35812	0.7247
Artificial model	12	0.67	0.3877	1.2259	0.2458

Initial preference *p*-values were calculated using binomial tests with a null hypothesis of 0.5. Overall preference *p*-values were calculated using one-sample *t*-tests (olfactory, visual + olfactory and artificial model cues) and sign tests (visual cue) with a null hypothesis of 0. * indicates *p* < 0.05.

### Lionfish prefer broad-scale, rather than fine-scale, habitat complexity

**Figure 1:**
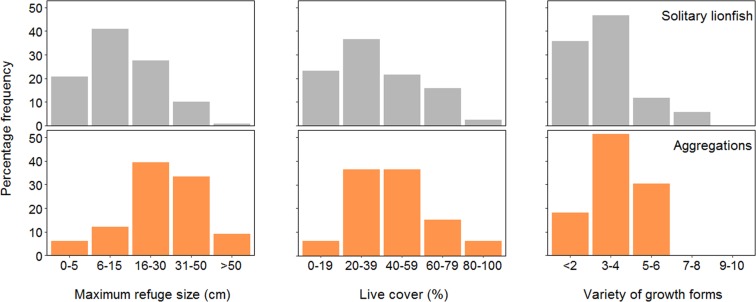
Habitat assessment scores for solitary lionfish and aggregations. The percentage frequency of lionfish in each habitat assessment score category is plotted for solitary lionfish (grey bars; *n* = 120) and aggregations (orange bars; *n* = 33).

Habitat assessment scores found that aggregating lionfish inhabited areas with significantly larger refuge sizes (*p* < 0.001), higher percentage live cover (*p* < 0.05) and a greater variety of growth forms (*p* < 0.05) than the areas inhabited by solitary lionfish (Fig. 1). There were no significant differences for rugosity or substratum height (*p* > 0.05 for both).

We also used 3D modelling to test for lionfish preferences towards habitat complexity. As there were no significant differences in the three complexity metrics (linear rugosity, vector dispersion and fractal dimension) between reef areas harbouring solitary lionfish and aggregations, these data sets were pooled to represent complexity with lionfish (lionfish quadrats) and compared with randomly selected areas of reef without lionfish (background quadrats).

**Figure 2:**
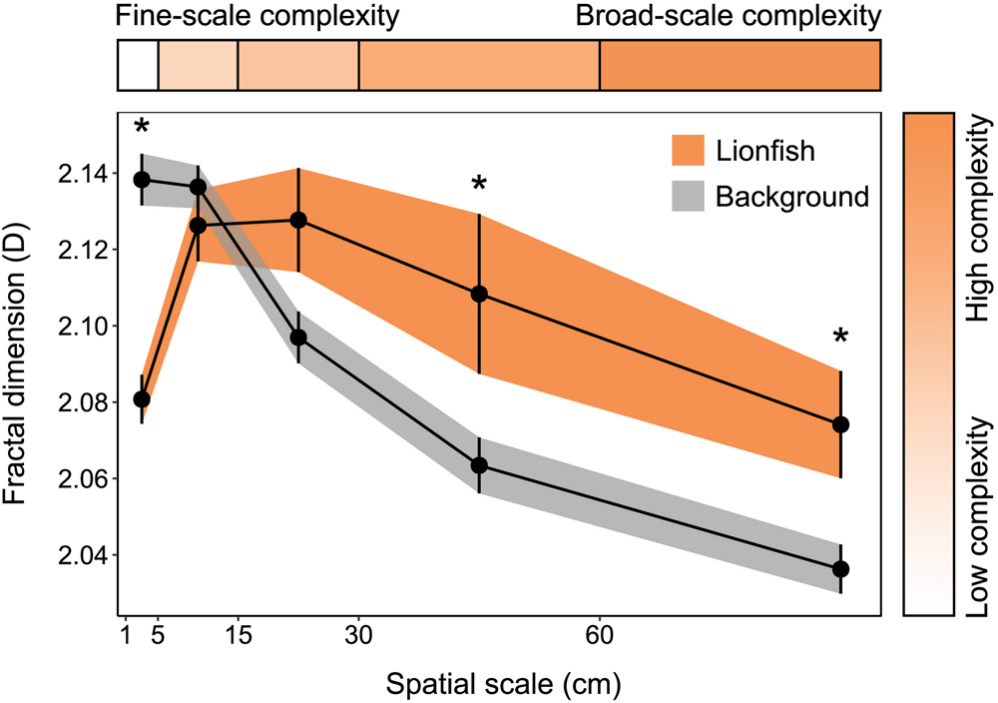
Fractal dimension at varying spatial scales. Fractal dimension values were calculated at five spatial scales: 1–5 cm, 5–15 cm, 15–30 cm, 30–60 cm and 60–120 cm. Means (±1 SE) are plotted at the midpoint of each spatial scale range for both lionfish quadrats (orange; *n* = 30) and background quadrats (grey; *n* = 44). * indicates *p* < 0.05.

Although there was no significant difference in linear rugosity between lionfish quadrats and background quadrats (*p* > 0.05; Table 2), lionfish were found in areas with significantly lower vector dispersion values (mean ± 1 SE; 0.144 ± 0.009 compared with 0.239 ± 0.007), indicating an association with low complexity at fine spatial scales (*p* < 0.001; Table 2). This is supported by assessment of fractal dimension across five spatial scale ranges (Table 2; Fig. 2). Lionfish quadrats were significantly less complex than background quadrats at the finest spatial scale (1–5 cm; *p* < 0.001). Conversely, lionfish quadrats were significantly more complex than background quadrats at the broadest spatial scales (30–60 cm and 60–120 cm; *p* < 0.05 for both). At intermediate spatial scales (5–15 cm and 15–30 cm) there was no significant difference. These results demonstrate an association with low complexity at fine spatial scales but high complexity at broad spatial scales.

**Table 2:** Tests of complexity between lionfish and background quadrats.

Complexity measure		Test statistic	*p*-value
Rugosity		−1.8179	0.07534
Vector dispersion		8.7092	<0.001*
Fractal dimension	1–5 cm	6.3722	<0.001*
	5–15 cm	0.9375	0.3528
	15–30 cm	−1.7142	0.09248
	30–60 cm	−2.2406	0.02899*
	60–120 cm	−2.5757	0.01255*

Complexity was compared between lionfish quadrats (n = 30) and background quadrats (n = 44). Rugosity was tested using a Welch’s *t*-test, vector dispersion was tested using a two-sample* t*-test and all fractal dimension spatial scales were tested using ranked Welch’s* t*-tests. * indicates p < 0.05.

## Discussion

Prior to this study, lionfish aggregation behaviour had only been observed anecdotally and little was known about its drivers. Through a combination of approaches and both *in situ* and *ex situ* data collection we have made a substantial contribution to this aspect of invasive lionfish research.

We found that aggregating individuals were larger than solitary individuals. Although larger female fish are often more fecund^35^, we found no difference in reproductive maturity between solitary and aggregating lionfish. This suggests that the aggregations observed during our study period were not for the purpose of spawning, unlike in other reef fish species^36,37^. Alternatively, aggregating lionfish may be larger because of higher growth rates, which may occur through cooperative hunting (which has been demonstrated to improve hunting success^38^) or because lionfish choose areas that maximise their individual hunting success. Cooperative hunting has been observed in another lionfish species, *Dendrochirus zebra*, in the laboratory^38^ and in *P. miles* in its native range^22^. However, in Mexico lionfish were more likely to be seen hunting when solitary than when in aggregations^39^. Although we found no difference in percentage body fat between aggregating and solitary individuals, we did find a difference in the value of Fulton’s condition factor, with aggregating individuals being heavier for their size. This difference may result from the benefits of cooperative hunting, or because aggregations form in areas where hunting success is higher.

Many fish that are found in groups exhibit social attraction^40,41^ but lionfish showed no preference for any of the cues tested here, both in initial reaction or overall preference. This is not unique; a lack of response to visual cues has been observed in other aggregating fish^27^. The lack of response might have been caused by differing personalities between individuals, resulting in differing motivation to aggregate. Several fish species show personality (e.g^42,43^) and personalities can influence social attraction^43^. Although we did not control for personality differences, the lionfish we caught may have been the boldest individuals^44^, thus minimising personality differences between individuals.

Lionfish did show a significant response in the olfactory treatment, however, their preference was for the control. This may have been due to the presence of waste products (e.g. ammonia^45^) in the olfactory cue, which are known to stimulate avoidance behaviour in other fish species^46^. Based on this result we suggest that future studies should use alternative methods of introducing olfactory cues, such as a lionfish behind an opaque, but water permeable, barrier.

Sound has a well-established role in reef fish behaviour^47,48^ and lionfish are known to produce sound^49^, therefore the use of acoustic cues for social attraction is possible. Acoustic cues were not standardised in our experiment therefore we cannot deduce whether lionfish use acoustic cues for social attraction. However, our findings do suggest that aggregating behaviour in invasive lionfish is not driven by visual or olfactory cues between conspecifics.

Previous studies provide mixed results on the association of lionfish with habitat complexity. Lionfish have been anecdotally reported from more complex habitat^10,16,17^ and shown to be associated with areas of greater hard coral cover and overhanging structures^50^. However, other studies have reported no relationship between habitat complexity and lionfish density or biomass^10,51,52^. By using more rigorous methods and quantifying multiple metrics of reef structure, we found a clear link between lionfish, aggregations and habitat complexity.

Firstly, our data show aggregations were associated with larger refuge sizes, higher live cover and a greater variety of growth forms. Preference of lionfish for these habitats may increase food availability; larger refuges can be associated with greater fish abundance^53^, increased coral cover may provide preferred habitat for larval settlement of prey species^54^ and a greater variety of growth forms can be associated with increased species richness^31^.

Lionfish habitat preference varied across different spatial scales. At finer scales (1–5 cm for fractal dimension and 1 cm for vector dispersion) lionfish were associated with lower complexity. Predatory fish may hunt less effectively in habitats that are complex at fine scales^55^, therefore lionfish may avoid high complexity at this scale to maximise hunting success. If this is indeed the case, lionfish hunting success may be increased because of reef flattening which is occurring throughout the Caribbean^56^. At broader scales (30–60 cm and 60–120 cm for fractal dimension) lionfish were associated with higher complexity that is often created by large crevices and overhangs. The erosion of broad-scale complexity as part of reef flattening may be less important to lionfish as artificial reefs such as shipwrecks and concrete structures can provide this type of complexity. This may explain why lionfish were found at higher density on artificial, rather than natural, reefs in the Gulf of Mexico^57^. Our study highlights the importance of considering spatial scale in assessments of species associations with habitat complexity. By creating “complexity signatures” using fractal dimension (Fig. 2) a much more detailed picture can be gained of how a species associates with both the quality and quantity of complexity^32^.

Caves and overhangs provide low light environments that may increase hunting success, given that lionfish prey capture rates have been shown to be higher on overcast than clear days^58^. The physical structure of caves and overhangs may also benefit lionfish hunting; lionfish have been observed herding prey towards vertical and concave surfaces before striking^58^.The inverse relationship between substratum height and availability of prey refugia^59^ suggests that these habitats often lack refuges for prey species. However, these habitats may provide shelter for certain prey and mutualist species. For example, overhangs provide shelter for *Gramma loreto*^60^, a fish that has been found in lionfish stomachs^61,62^ but that is also a potential lionfish cleaner^16^.

Based on these findings, we propose that aggregations of invasive lionfish are coincidental and form when multiple lionfish are attracted to the same area of reef, rather than through visual or olfactory attraction to conspecifics. This attraction to broad-scale over fine-scale complexity is likely to be driven by a search for maximised hunting efficiency, characterised by reduced refugia for prey items, leading multiple lionfish to congregate on areas of reef where feeding is easiest.

This knowledge could be used to significantly improve the efficiency of culling efforts. Our results can be used to inform the design of artificial aggregation devices or to improve existing lionfish traps that already involve some complexity^15^, but which could benefit from incorporating broad-scale crevices and minimising fine-scale refugia. Artificial aggregation devices and traps could in turn extend the depth limit of culling efforts to exploit the significant populations found on mesophotic reefs^12,13^, which are believed to include the most reproductively active individuals^12^. Only through improved understanding of the behaviour and ecology of this widespread invader will managers have the upper hand. By expanding and improving culling efforts using the findings of this study, a long-term solution will be one step closer.

## Methods

### Study sites

**Figure 3:**
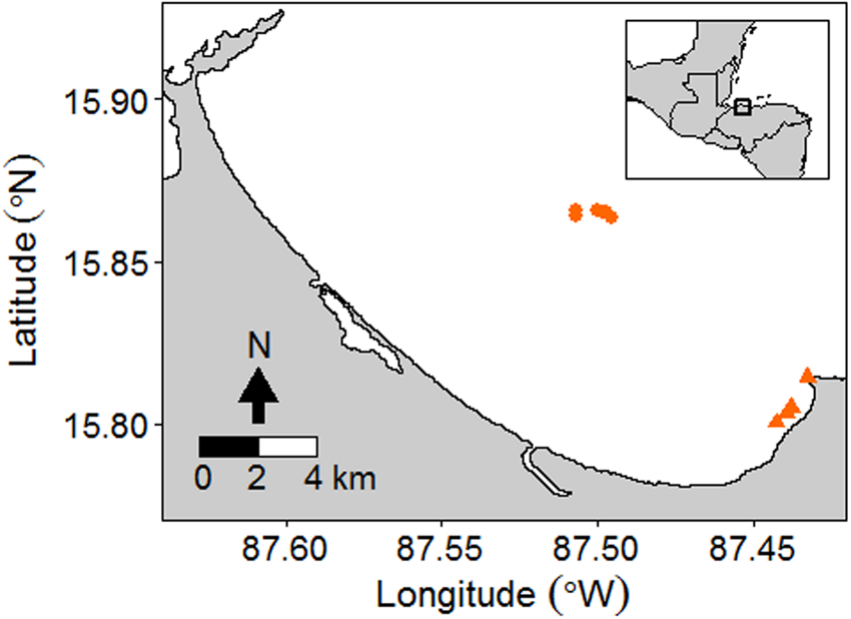
Map of our study sites in Honduras. Population structure data and habitat assessment scores were recorded from all 10 sites. Triangles mark the four sites on the La Ensenada reef system where lionfish were collected live for laboratory social attraction experiments. Circles mark the six sites on the Banco Capiro reef system where lionfish were culled and 3D modelling was carried out. The location of Tela bay, Honduras within central America is shown on the inset map.

All data were collected from reefs in the mainland Tela Bay, Honduras (Fig. 3; Supplementary Table 1) between June and August 2017 using open-circuit SCUBA. Lionfish were culled for dissection at six sites on the offshore reef system of Banco Capiro at depths between 10–18 m (see Bodmer *et al*.^63^ for a general site description). Lionfish were collected alive for use in laboratory social attraction experiments from four sites on the shallow nearshore reef system of La Ensenada at depths <10 m. A recent genetic study suggests invasive lionfish in Honduras are likely to all be *P. volitans*^64^, and so we assumed this to be the case. However, we did not identify them to species level and so will refer to them simply as lionfish hereafter.

### *In situ* population structure surveys and *ex situ* dissections

Roving diver surveys were conducted, and any lionfish encountered deeper than 10 m were culled using pole spears before being transferred into a ZooKeeper container (ZooKeeper LLC, USA) for transport back to the laboratory. Approximate size, supra-ocular tentacle type and distinguishing features (e.g. unusual patterning) were noted *in situ* to enable later identification of individuals. Depth of capture and, if aggregating, the number of individuals within the aggregation were also recorded. An aggregation was defined here as two or more individual lionfish being found within approximately four body lengths of one another^24^.

On return to the laboratory, culled lionfish were refrigerated and dissected within 12 hours. Total length was recorded to the nearest mm from the tip of the snout to the longest point of the tail. Percentage body fat, which was used as a measure of feeding success, was calculated as fat weight (to the nearest 0.1 g) divided by body weight (to the nearest g), then multiplied by 100. Fat weight was obtained by removing all fat deposits from within the gut cavity and weighing these using an electronic balance. Fulton’s condition factor, which was also used as a measure of feeding success, was calculated as body weight divided by total length cubed^65,66^, with higher values indicating that fish were heavier for their size. Macroscopic analysis of the gonads was used to determine sex^67^. Lionfish reproduce throughout the year^68^, which enabled us to measure reproductive maturity during our study. Reproductive maturity of males and females was measured using the gonadosomatic index^68^, which was calculated as gonad weight (to the nearest 0.1 g) divided by body weight and then multiplied by 100. Dissection methods followed Green *et al*.^67^.

### Laboratory visual and olfactory attraction experiments

#### Lionfish capture and experimental setup

A total of 58 lionfish were captured alive using hand nets and transferred to large plastic dry-bags, before being slowly brought to the surface. Lionfish were transported inside the dry-bags to the laboratory by 15-minute boat ride. On arrival at the laboratory lionfish were immediately transferred to the holding tank. Live collections were restricted to <10 m to avoid barotrauma injuries. Lionfish ranged in total length from 4.7–29.5 cm with a mean (±1 SE) of 17.0 (±0.88) cm. Each lionfish was used once to explore one of (i) visual cues (*n* = 15), (ii) olfactory cues (*n* = 13), (iii) visual + olfactory cues (*n* = 18) or (iv) artificial model cues (*n* = 12). Post-trial, some lionfish were used as stimulus fish for up to two trials. Stimulus fish were never re-used as test fish.

The holding and trial tanks measured 245 × 56 × 24 cm (length x width x depth) and were filled with unfiltered natural seawater to a depth of 16 cm. Water in the tanks was maintained at ambient temperature (29 °C). Water changes were conducted at 100% every 24 hours in the holding tank, and 100% between each trial in the trial tank using freshly collected seawater (which was assumed to be constant in aeration and quality). No lionfish were present in the tanks during water changes. Tanks were naturally lit by large windows and thus followed the ambient light:dark cycle. All experiments were conducted during daylight hours between 6:00 am and 6:00 pm. During the trials two artificial lights (10 watt LED lamps) were used above the trial tank to account for shading by the building structure and ensure standardisation of lighting between all trials.

Lionfish were kept in the holding tank for an acclimatisation period of 24 hours during which they were starved to ensure similar hunger levels between individuals^69^. A maximum of six lionfish were maintained in the holding tank at any time. The holding tank was fitted with a Tetra whisper power filter (Spectrum Brands Inc., USA) and Aqua Culture air pump (Walmart Stores Inc., USA) to ensure the water remained clean and aerated.

**Figure 4:**
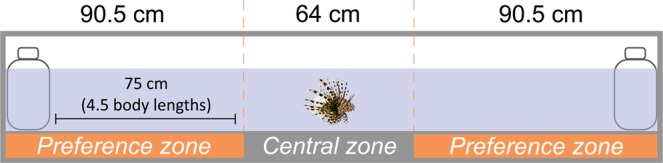
Tank set-up for laboratory social attraction experiments. The tank was divided into two preference zones (stimulus and control) and a central zone. Dotted lines mark boundaries between zones, which were marked by placing tape across the top of the tank. A GoPro Hero5 camera (GoPro Inc., USA) was mounted on the ceiling 1 m above the centre of the tank. Diagram is not drawn to scale. Lionfish photo ©iStock.com/GlobalP.

We used one trial tank that was divided into two preference zones of 90.5 cm (one at either end of the tank) and a central zone of 64 cm, by placing tape across the top of the tank (Fig. 4). The preference zones encompassed a 15.5 cm diameter stimulus container plus approximately 4.5 body lengths distance from the stimulus container, based on the average total length of 17.0 cm for lionfish in this study. This distance encompassed the four body length distance used to define fish aggregations^24^ to ensure that, if present, aggregation behaviour was detected. The tank was surrounded by opaque white plastic to prevent interference from external stimuli^70^.

#### Experimental design

**Figure 5:**
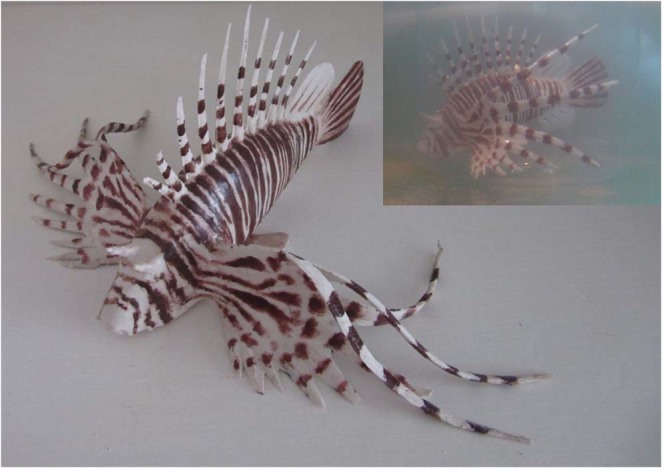
Artificial model lionfish used in the laboratory social attraction experiments. The model was moulded from aquarium grade silicone using a custom-made mould created photogrammetrically using video footage of a live lionfish. The model measures 15 cm total length. Underwater the pectoral fins spread out to give a more realistic appearance (inset photograph).

Stimuli were added to one end of the trial tank based on the following: (i) live lionfish and water from the trial tank added to a submerged transparent plastic container (visual cue), (ii) water from the holding tank added to a submerged transparent plastic container with 30 evenly spaced 4 mm diameter holes (olfactory cue), (iii) live lionfish and water from the trial tank added to a submerged transparent plastic container with 30 evenly spaced 4 mm diameter holes (visual + olfactory cue), and (iv) 3D printed lionfish model (Fig. 5) and water from the trial tank added to a submerged transparent plastic container (artificial model cue). A control was placed at the opposite end of the tank, comprising water from the trial tank added to a submerged transparent plastic container without holes (visual and artificial model controls) or with 30 evenly spaced 4 mm diameter holes (olfactory and visual + olfactory controls). The end of the tank containing the stimulus/control was randomised between trials and the order of trials was randomised between days.

A randomly chosen lionfish was placed in a 30 cm diameter mesh acclimatisation cylinder in the centre of the tank. After a ten minute acclimatisation period^29^ (which allowed the lionfish to acclimatise to the water, stimuli and artificial lighting), the cylinder was lifted and the lionfish was filmed for a trial period of ten minutes^29^ using a GoPro Hero5 camera (GoPro Inc., USA) mounted 1 m above the centre of the tank. Subsequently, the videos were analysed and the position of the lionfish (stimulus, control or central zone) recorded every second for the duration of the trial, as well as which preference zone (stimulus or control) was entered first. Position was determined as the zone that the tip of the lionfish’s snout was in. Overall preference was calculated as time spent in the stimulus zone minus time spent in the control zone.

Once lionfish had been used in a trial they were humanely culled by cervical transection, followed by pithing to destroy the brain tissue^71^. These lionfish were subsequently dissected as described earlier, except that fat weight was not recorded.

### Habitat preference and habitat complexity

Whenever lionfish were encountered on the reef, a 1 × 1 m quadrat was placed over the reef centred on the position of the individual lionfish or the centre of the aggregation. Habitat assessment scores were then performed following Gratwicke and Speight^31^, whereby five categories of structure were estimated visually on a five-point scale. Maximum substratum height, refuge size and variety of growth forms were recorded, along with average live cover (percentage cover of sessile organisms such as live corals, macroalgae and sponges) and rugosity scores for each quadrat. Over the course of the study 153 quadrats were assessed. Only one data point for complexity was taken per aggregation.

In addition, 30 large quadrats (2 × 2 m) were placed on reef areas where lionfish were encountered (23 solitary and 7 aggregations). These quadrats were filmed in a lawnmower pattern, approximately 0.5 m above the benthos, using a GoPro Hero3 camera (GoPro Inc., USA). Areas of reef without lionfish present were also sampled to give a background average for the reef system. Transects were laid at randomly chosen directions from each mooring line and quadrats were placed at regular intervals along each transect to give a total of 44 background quadrats. The resulting footage was converted into stills and used to render 3D models in Agisoft Photoscan (Agisoft LLC, Russia), before being analysed for habitat complexity using Rhinoceros 3D (Robert McNeel and Associates, USA), following the method of Young *et al*.^32^. Habitat complexity was measured using three metrics: linear rugosity (2 cm resolution), vector dispersion (1 cm resolution) and fractal dimension. Fractal dimension was measured at five spatial resolutions (1–5 cm, 5–15 cm, 15–30 cm, 30–60 cm and 60–120 cm) to explore patterns between lionfish habitat preference and habitat complexity at varying spatial scales.

### Statistical analyses

All tests were two-tailed with an a-priori significance level of 0.05 and were conducted in R version 3.4.2 (R Core Team, Austria). Prior to analysis, datasets were checked for normality using a Shapiro-Wilk test, and groups were checked for homogeneity of variance using Fisher’s *F* test. When the assumption of normality was violated, transformation was attempted. When data transformation did not normalise the data, a non-parametric test was conducted on the original data.

For the social attraction experiments, initial preference was tested using binomial tests and overall preference was tested using one-sample *t*-tests or sign tests. For tests between two groups (lionfish quadrats and background quadrats or solitary lionfish and aggregations), two-sample *t*-tests were used for continuous, normally distributed data, whilst two-proportions *Z*-tests were used for proportions. For continuous data that violated the normality assumptions, and for ordinal data, Mann-Whitney *U*-tests were conducted. Data that violated both normality and homogeneity of variance assumptions were analysed using a ranked Welch’s *t*-test^72,73^.

### Ethics statement

Lionfish culling was carried out in accordance with the American Veterinary Medical Association Guidelines for the Euthanasia of Animals^71^. All experimental protocols were approved by the University of Oxford Animal Welfare and Ethical Review Body. No permits were required to collect lionfish, however, a research permit to allow this work to be conducted in Honduras was obtained from the Instituto de Conservacion Forestal Honduras (permit number: DE-MP-081-2017).

## Supplementary information

Supplementary Table 1

## Data Availability

The data that support the findings of this study are available from the corresponding author upon reasonable request.
