# Supplementary material for: Aggregating behaviour in invasive Caribbean lionfish is driven by habitat complexity
Source: Sci Rep. 2019 Jan 28;9:783. doi: 10.1038/s41598-018-37459-w (PMC6349842; doi:10.1038/s41598-018-37459-w)
Supplement: Supplementary file 1 — Supplementary Table 1 [file 41598_2018_37459_MOESM1_ESM.docx]

**Aggregating behaviour in invasive Caribbean lionfish is driven by habitat complexity**

Hunt C.L^1,2^*, Kelly G.R^2,3^, Windmill H^2^, Curtis-Quick J^4^, Conlon H^2^ , Bodmer M.D.V^2,5^, Rogers A.D^1†^, Exton D.A^2†^

^1^ Department of Zoology, University of Oxford, John Krebs Field Station, Wytham, OX2 8QJ, UK

^2^ Operation Wallacea, Wallace House, Old Bolingbroke, Spilsby, Lincolnshire, PE23 4EX, UK

^3^ Department of Ocean and Earth Science, University of Southampton, National Oceanography Centre, SO14 3ZH, UK

^4^ School of Earth and Ocean Sciences, University of Cardiff, Main Building, Park Place, CF10 3AT, UK

^5^School of Environment, Earth and Ecosystem Sciences, The Open University, Walton Hall, Milton Keynes, MK7 6AA, UK

* Corresponding author: [christina.hunt@zoo.ox.ac.uk](mailto:christina.hunt@zoo.ox.ac.uk)

^†^These authors contributed equally and are joint last authors

Supplementary Table 1

**Study site co-ordinates.** Co-ordinates of our 10 study sites within Tela bay, Honduras.

| Reef system | Site name | Latitude (^o^N) | Longitude (^o^W) |
| --- | --- | --- | --- |
| Banco Capiro | Aldrid’s | 15.86496 | 87.49841 |
|  | Butterfingers | 15.86351 | 87.49525 |
|  | Canyon | 15.86414 | 87.50662 |
|  | Kisci’s garden | 15.86550 | 87.50017 |
|  | Mushroom mountain | 15.86497 | 87.4973 |
|  | Rotunda | 15.86561 | 87.50680 |
| La Ensenada | Acuario | 15.81423 | 87.43244 |
|  | Becky’s choice | 15.80494 | 87.43768 |
|  | Nelly’s island | 15.80017 | 87.44264 |
|  | Palm view one | 15.80339 | 87.43922 |
